# Feasibility and acceptability of smartphone technology for patients to self-record vital signs in the emergency department (The FacED Study): A study protocol

**DOI:** 10.1371/journal.pone.0326013

**Published:** 2026-06-30

**Authors:** Jared Charlton-Webb, Kathryn Willis, Gabriel Jones, Sarah White, Caspar Norris, Manisha Bageya, Heather Jarman

**Affiliations:** 1 Emergency Department Collaborative Research Group, St. George’s University Hospitals NHS Foundation Trust, London, United Kingdom; 2 Population Health Research Institute, School of Health and Medical Sciences, City St. Georges, University of London, London, United Kingdom; Coventry University, UNITED KINGDOM OF GREAT BRITAIN AND NORTHERN IRELAND

## Abstract

Overcrowding and high nursing workload in urgent and emergency care (UEC) settings frequently lead to incomplete or omitted measurement of patient vital signs during initial assessment and monitoring, with potential negative consequences for patient outcomes. Contactless camera‑based photoplethysmography (PPG) software, which allows patients to record a 30‑second smartphone video to obtain their own vital signs, may help reduce nursing workload, support prioritisation of clinical tasks, and decrease waiting times for triage. This study will evaluate the feasibility and acceptability of this technology in situ across three UEC services at St George’s University Hospitals NHS Foundation Trust. A total of 1,500 patients will undergo both manual and smartphone‑based vital‑sign measurement, and feasibility will be assessed using a post‑measurement survey. Acceptability among triage staff will be evaluated using a short survey of 20–40 clinicians, and broader patient acceptability of digital health technologies in UEC settings will be explored through a questionnaire administered to 10,000 patients. Informed consent will be obtained before participation in any study procedures. Findings will be disseminated through peer‑reviewed publications and conference presentations and will inform future research on digital vital‑sign monitoring in UEC environments.

## Introduction

One of the biggest issues facing Urgent and Emergency Care Settings (UEC) internationally is crowding, defined as when “the demands on an [emergency department] ED exceed the capacity” [1]. Crowding is known to lead to long wait times, delays for assessment, increased patient morbidity and poor health outcomes [2,3]. Whilst several strategies to reduce delays to assessment have been proposed, including frontloading triage teams and rapid assessment models [1,4], the issue of ongoing monitoring and reassessment of patients in crowded waiting rooms has yet to be clearly tackled.

A key component of patient assessment and reassessment in UECs is the recording of vital signs. Patient vital signs include heart rate, blood pressure, oxygen saturation, temperature, and respiratory rate. In UEC vital signs are recorded at least once, firstly as part of an initial assessment for patients’ presenting with illness conditions [5], and if needed, to monitor for clinical deterioration or improvements [6]. Despite the need to complete timely and regular recording of vital signs there is evidence that factors such as heavy nursing workload and ED crowding leads to incomplete or complete omission of recording [7,8].

One potential solution that may ease nursing workload caused by high numbers of patients and low throughput is the implementation of patient self-recorded vital signs using smartphone camera-based photoplethysmography (PPG). This may have utility for both initial assessment and ongoing monitoring of vital signs in stable patients, reducing the duration of the triage assessment and allowing nursing staff to focus on higher order tasks. Previous literature has implied high acceptability among ED patients with regards to using mobile devices to access and enter their personal and healthcare information. [9,10].

Camera PPG software to measure vital signs has been explored in various healthcare settings including cardiology [11], geriatrics [12], paediatrics [13], and intensive care [14]. Research within UEC settings reveals that camera PPG software could prove successful within ED triage [15,16], however previous work has focused on piloting the accuracy of the application [12,15,16] using small sample sizes and has not been widely trialled in a real-life uncontrolled scenario with a large population [17].

## Aims and objectives

The main aim of the study is to determine the feasibility and acceptability of patient facial self-recorded vital signs for adult walk-in patients in the ED. The objectives are to:

- Explore the performance of facial recording of vital signs compared to standard methods
- Determine clinician acceptability of facial recording of vital signs in the ED

A secondary objective is to establish whether patients in urgent and emergency care settings are be interested in, or willing to use, mobile health technologies in their care.

Although reductions in crowding, improved utilisation of clinical staff time, and improved completeness of vital‑sign documentation are potential downstream benefits of patient‑recorded vital signs, these outcomes will not be evaluated within this feasibility protocol. The current study will focus on assessing usability, acceptability, feasibility, and preliminary agreement between smartphone‑derived and standard measurements. Evaluation of system‑level impacts will form part of future research.

## Methods

### Study design

This is a prospective, multi-site study comprising three inter-related work packages: feasibility and acceptability of self-recording of vital signs by patients (WP1), staff acceptability (WP2), and patient acceptability of mobile health technology in their care (WP3). The protocol was developed according to the Strengthening of Reporting of Observational Studies in Epidemiology (STROBE) statement [18].

Although reductions in crowding, improved utilisation of clinical staff time, and improved completeness of vital‑sign documentation are potential downstream benefits of patient‑recorded vital signs, these outcomes will not be evaluated within this feasibility protocol. The current study will focus on assessing usability, acceptability, feasibility, and preliminary agreement between smartphone‑derived and standard measurements. Evaluation of system‑level impacts will form part of future research.

## Setting

For WP1 and WP2, recruitment will take place in the largest of 3 EDs in George’s, Epsom and St Helier Hospital Group in London. For WP3 (large scale survey) all 3 EDs will recruit participants. Recruitment will take place from 01 June 2025–31 December 2026.

## Participants

Participants will be identified by members of the clinical or research teams working in any of the sites. Potential participants will be screened using electronic medical records to exclude patients in need of urgent medical assessment prior to approach. Screened patients will be approached by members of the research team and assessed against the eligibility criteria (Table 1). For WP2 (clinical staff questionnaire) we will email eligible staff members to request participation.

**Table 1:** Eligibility criteria for the FacED feasibility study.

	WP1 (patients)	WP2 (staff)	WP3 (patients
Inclusion criteria	• ‘Walk-in’ adult patients ≥ 18yrs in ED or UTC • Has access to own smartphone • Willing and able to give informed consent for participation in the study	• Clinical staff (e.g., nurse, doctor) working in triage or assessment area of either ED or UTC	• ‘Walk-in’ adult patients ≥ 18yrs in ED or UTC Has access to own smartphone Willing and able to give informed consent for participation in the study
Exclusion criteria	• Clinical condition warranting immediate medical attention (e.g., triage category 1)	• Unwilling to complete questionnaire • Clinical staff not involved in triage or assessment	• Clinical condition warranting immediate medical attention

For WP3, clinically stable participants will be approached in the ED whilst waiting to be seen. Posters with QR codes in waiting rooms at participating sites will also be used to allow patients to access the survey directly.

### Informed consent procedures

Written informed consent will be obtained from all participants in WP1 prior to completing any study‑related procedures. For WP2 and WP3, consent will be obtained via a self‑completed electronic process embedded within the online questionnaires. Participants will review a digital information sheet and will confirm each mandatory consent statement before proceeding. No verbal consent procedures will be used. Research staff will not be able to view or identify individual respondents for WP2 or WP3.

## Study size

WP1: the smartphone vital signs recording work package will use convenience sampling. Agreement between smartphone‑derived vital signs and standard clinician‑measured vital signs will be assessed using the intraclass correlation coefficient (ICC). The ICC is the recommended metric when evaluating agreement between two continuous measurement methods obtained under similar conditions, as it quantifies the proportion of total variability that is due to true agreement rather than measurement error. The sample size for WP1 is based on achieving a precise estimate of the ICC, assuming an expected ICC of 0.75 for agreement between smartphone and standard measurements, a sample of 1,178 participants will be required to produce a 95% confidence interval with a width of 0.025. By recruiting 1,500 participants, we will allow for attrition and missing data while retaining sufficient precision in the ICC estimate to plan future evaluative studies.

The purpose of this study is to determine feasibility and acceptability; however, an estimate of measurement agreement is necessary to inform future definitive studies. Therefore, the ICC will not function as a co‑primary outcome, but rather as an important secondary metric to understand the likely performance of the technology under real‑world emergency care conditions.

WP2: uses a non-probability convenience sample of clinical staff working in the assessment areas of each of the sites. We aim to obtain a representative clinical staff (by profession and experience) and will recruit between 20 and 40 members of staff.

WP3: uses a non-probability convenience sample of patients presenting to the ED over the recruitment period. There is no sample size calculation for the survey on digital health use in the ED patients. Reason for attendance is not a factor in inclusion and based on previous work using similar recruitment methods we anticipate that 10,000 participants will be recruited over the study duration.

## Variables

The outcome variables to assess the study aims are as follows:

- Feasibility: defined as the proportion of patients consented who were able to complete the vital signs recording using their smartphones. Feasibility also includes the proportion of participants who were able to complete the process without staff assistance and completeness of recorded measurements.
- Acceptability of self-recorded vital signs (patients): affinity for technology use – how comfortable, confident, and willing participants are when using digital or technological tools; and acceptability and patient experience of use of PPG application
- Performance: reliability of the platform to accurately measure the vital signs (blood pressure, heart rate, respiratory rate, oxygen saturation) recorded using a web-based PPG application compared to near simultaneous measurement of vital signs using a standard monitor. The Fitzpatrick skin‑tone scale will be used to support subgroup analyses of ICC to explore whether measurement agreement varies by skin tone [19].
- Acceptability (clinical staff): electronic survey of personal technical affinity and a usability evaluation of the patient-recorded vital signs

## Data sources/measurement

The data collected in this study is anonymised and all participants will be assigned a unique identification (ID) number.

**Fig 1:**
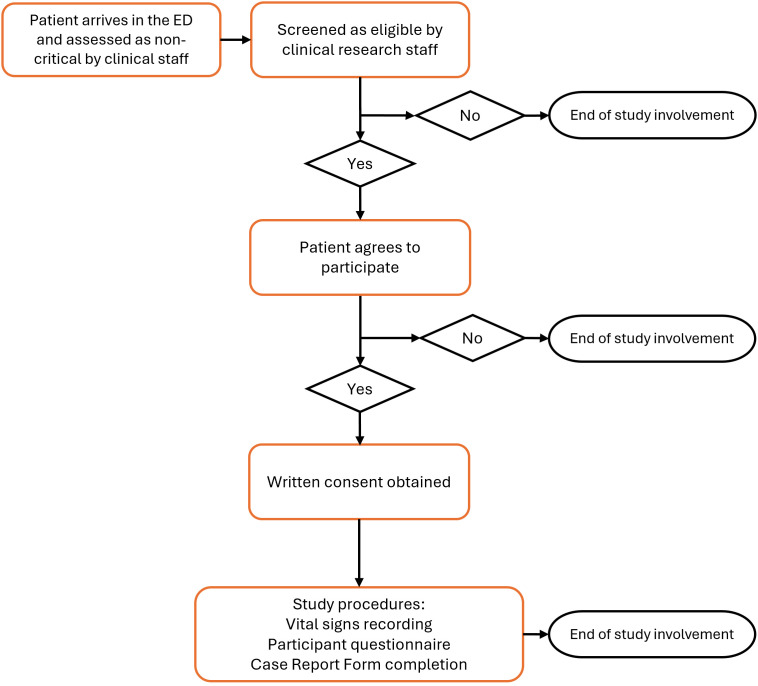
Study flow diagram depicting the recruitment process for WP1.

For WP1, participants will be asked to complete two types of data collection. A flow chart for WP1 can be found in Fig 1.

- A clinical research nurse will support the patient through the data collection phases if necessary. Completion of their vital signs using PPG technology on their smartphone accessed through a QR code link on a webpage. The webpage will not capture any personal information, and all data is removed once the participant leaves the website. Participants will be guided through the capture of their vital signs by a research nurse if needed. Clinical or research staff will near-simultaneously complete the patients vital signs using traditional methods. To ensure comparability, all clinical staff measuring vital signs using standard equipment will receive study‑specific training on timing, device placement, and measurement procedures. Near‑simultaneous measurement will be defined as occurring within two minutes of the PPG recording. Staff will record the timestamp of each measurement to verify this.
- Post-PPG recording participant acceptability data will be captured electronically using an online questionnaire. Participants will be able to complete the survey on their personal devices, following a linked QR code or can be provided with a handheld electronic device. The electronic survey contains the invitation to participate letter (participant information sheet), consent form and the questionnaire. Advantages of the electronic format include ease of administration, ease of data collection and reduced environmental impact versus the use of paper. The questionnaire has been designed to meet the study objectives, drawing from previous literature on digital technology acceptability and technical affinity. The questionnaire is comprised of 17 questions. There are 4 demographic questions, Fitzpatrick skin tone scale, and smartphone operating system. 8 questions including The Single Ease Question (SEQ) and System Usability Scale (SUS) [19–21] will be used to measure the usability of the software, and 5 questions will measure self-reported technical affinity [22] . The survey should take no longer than 5 minutes for each participant to complete, and participants will be notified of this at the start of the survey.
- Participant deprivation index and ED length of stay information will be captured from the hospital electronic patient record (EPR) by the research team and linked to the patient acceptability data following consent.

To determine levels of clinical staff acceptability of the use of PPG technology (WP2), participants will complete an electronic questionnaire designed to capture workplace demographics (profession and seniority), 5 questions will measure self-reported technical affinity [22] and 10 questions to determine the perceived usefulness of the PPG technology on their practice [23].

Finally, to establish whether patients in urgent and emergency care settings are be interested in, or willing to use, mobile health technologies in their care (WP3) we will collect data on age, gender, ethnicity, 1st language, educational attainment level and smartphone ownership. Self-reported technical affinity will be measured with 5 questions [22] and rating questions on areas where participants feel it is acceptable to use digital health technologies as part of their care. It will take participants approximately 15 minutes to complete.

## Bias

Bias will be minimised in the study by collecting data through standardised reporting and assessment of measures and using a recognised evaluation questionnaires tool. The use of routine clinical measurements as the comparator may introduce bias due to inter‑observer variation and differences in training or technique. Standardisation procedures and training will be implemented to minimise this variability.

Exclusion of participants without English proficiency may limit generalisability and may disproportionately affect groups at higher risk of digital marginalisation. This issue will be explored in future work which will include language accessibility considerations in digital triage pathways.

## Analysis and statistical methods

The electronic surveys will be hosted on the REDCap Platform under a password-protected personal account held only by the Chief Investigator and study team. REDCap provides compliance with General Data Protection Regulations and is ISO 27001 compliant. A link to the REDCap Data protection policy is provided within the participant information sheet.

Descriptive statistics will be used to characterise participants by age, gender, highest educational attainment, IMD and ethnicity. Feasibility will be reported with a percentage and 95% CI. Acceptability to patients as measured by SEQ and overall, SUS score will be summarised, using mean and standard deviation (SD). The association between acceptability to patients and age, educational attainment, IMD and ethnicity will be assessed using regression methods. Descriptive statistics will be used to summarise the findings of the acceptability to clinical staff survey.

Performance of the app will be assessed using the intraclass correlation coefficient (ICC) for the whole sample and for a priori defined subgroups. ICC’s take values between 0 and 1 where, 0 implies no agreement and 1 is perfect agreement and represents the proportion of the total variability in the observations that is due to the differences between pairs [24] . A priori subgroups for exploratory agreement analyses will include age, sex, Fitzpatrick skin tone, and Index of Multiple Deprivation category. These subgroup analyses will be descriptive and hypothesis‑generating only. The study is not powered for between‑group comparisons, and no adjustments for multiple testing will be made. Subgroup ICCs will be estimated using bootstrapped confidence intervals to provide stable descriptive estimates for planning future studies.

## Patient and Public Involvement (PPI)

Effective patient and public involvement are of key importance to providing safe and effective emergency care. Discussions with ED service users informed the study design. Feedback emphasized the importance of clear instructions for internet application use and the brevity of questionnaires.

## Ethics and dissemination

The study obtained UK NHS ethical approval by the London – Surrey Research Ethics Committee (REC number 25/PR/0222) on 4^th^ April 2025. Informed consent will be obtained prior to participants undergoing any activities that are specifically for the purposes of the study. Consent for WP1 will be taken electronically by a member of the research team. For WP2 and WP3 consent is ‘self-completed’ online and requires no input from the clinical or research teams. Research staff will not be aware of who has taken part and will not be able to identify any participants. There is no follow-up or other direct contact by research staff.

Findings from this research will be shared and discussed with the study steering committee in preparation of wider dissemination through journals and conferences.

## References

[1] Royal College of Emergency Medicine. The management of emergency department crowding. 2024. https://rcem.ac.uk/wp-content/uploads/2024/01/RCEM_Crowding_Guidance_Jan_2024_final.pdf

[2] Mackway-Jones K, Marsden J, Windle J. Emergency Triage: Manchester Triage Group. 3rd ed. 2013. 10.7748/en2013.07.21.4.11.s11 23901863

[3] Royal College of Physicians. National Early Warning Score (NEWS) 2: Standardising the assessment of acute-illness severity in the NHS. 2017. www.rcplondon.ac.uk

[4] Redfern OC, Griffiths P, Maruotti A, Recio Saucedo A, Smith GB, Missed Care Study Group. The association between nurse staffing levels and the timeliness of vital signs monitoring: a retrospective observational study in the UK. BMJ Open. 2019;9(9):e032157. doi: 10.1136/bmjopen-2019-032157 31562161 PMC6773325

[5] van der Linden MC, Meester BEAM, van der Linden N. Emergency department crowding affects triage processes. Int Emerg Nurs. 2016;29:27–31. doi: 10.1016/j.ienj.2016.02.003 26970907

[6] Selvaraju V, Spicher N, Wang J, Ganapathy N, Warnecke JM, Leonhardt S, et al. Continuous monitoring of vital signs using cameras: a systematic review. Sensors (Basel). 2022;22(11):4097. doi: 10.3390/s22114097 35684717 PMC9185528

[7] Bautista M, Cave D, Downey C, Bentham JR, Jayne D. Clinical applications of contactless photoplethysmography for vital signs monitoring in pediatrics: a systematic review and meta-analysis. J Clin Transl Sci. 2023;7(1):e144. doi: 10.1017/cts.2023.557 37396820 PMC10310860

[8] Yu X, Laurentius T, Bollheimer C, Leonhardt S, Antink CH. Noncontact monitoring of heart rate and heart rate variability in geriatric patients using photoplethysmography imaging. IEEE J Biomed Health Inform. 2021;25(5):1781–92. doi: 10.1109/JBHI.2020.3018394 32816681

[9] Capraro GA, Balmaekers B, den Brinker AC, Rocque M, DePina Y, Schiavo MW, et al. Contactless vital signs acquisition using video photoplethysmography, motion analysis and passive infrared thermography devices during emergency department walk-in triage in pandemic conditions. J Emerg Med. 2022;63(1):115–29. doi: 10.1016/j.jemermed.2022.06.001 35940984

[10] Kobayashi L, Chuck CC, Kim CK, Luchette KR, Oster A, Merck DL, et al. Comparison of video photoplethysmography, video motion analysis, and passive infrared thermography against traditional contact methods for acquiring vital signs in emergency department populations. In: 2023 IEEE 14th Annual Ubiquitous Computing, Electronics & Mobile Communication Conference (UEMCON). 2023. p. 0134–41. doi: 10.1109/uemcon59035.2023.10316088

[11] Mather JD, Hayes LD, Mair JL, Sculthorpe NF. Validity of resting heart rate derived from contact-based smartphone photoplethysmography compared with electrocardiography: a scoping review and checklist for optimal acquisition and reporting. Front Digit Health. 2024;6:1326511. doi: 10.3389/fdgth.2024.1326511 38486919 PMC10937558

[12] UK mobile phone statistics 2024 - stats report. [cited 2025 Feb 28]. https://www.uswitch.com/mobiles/studies/mobile-statistics/

[13] Kim E, Torous J, Horng S, Grossestreuer AV, Rodriguez J, Lee T, et al. Mobile device ownership among emergency department patients. Int J Med Inform. 2019;126:114–7. doi: 10.1016/j.ijmedinf.2019.03.020 31029252

[14] Goldfine CE, Knapp A, Goodman GR, Hasdianda MA, Huang H, Marshall AD, et al. Media and technology usage and attitudes in emergency department patients. Front Digit Health. 2022;4:894683. doi: 10.3389/fdgth.2022.894683 36386045 PMC9659569

[15] Brooke J. SUS: a ‘Quick and Dirty’ usability scale. Usability evaluation in industry. CRC Press; 1996. p. 189–95.

[16] Doll WJ, Xia W, Torkzadeh G. A confirmatory factor analysis of the end-user computing satisfaction instrument. Doll WJ, Xia W, Torkzadeh G, editors. MIS Q. 1994;18(4):461. doi: 10.2307/249524

[17] Fritsch SJ, Blankenheim A, Wahl A, Hetfeld P, Maassen O, Deffge S. Attitudes and perception of artificial intelligence in healthcare: a cross-sectional survey among patients. Digit Health. 2022;8. doi: 10.1177/20552076221116772 PMC9380417 35983102

[18] Bonett DG. Sample size requirements for estimating intraclass correlations with desired precision. Stat Med. 2002;21(9):1331–5. doi: 10.1002/sim.1108 12111881

[19] Fitzpatrick TB. The validity and practicality of sun-reactive skin types I through VI. Arch Dermatol. 1988;124(6):869-71. doi: 10.1001/archderm.1988.01670060015008 3377516

[20] Doll WJ, Xia W, Torkzadeh G. A confirmatory factor analysis of the end-user computing satisfaction instrument. MIS Q. 1994;18(4):461. doi: 10.2307/249524

[21] Brooke J. SUS: A ‘quick and dirty’ usability scale. In: Jordan PW, Thomas B, Weerdmeester BA, McClelland IL, editors. Usability evaluation in industry. Boca Raton: CRC Press; 1996. p. 189-195. doi: 10.1201/9781498710411-35

[22] Fritsch SJ, Blankenheim A, Wahl A, et al. Attitudes and perception of artificial intelligence in healthcare: a cross-sectional survey among patients. Digit Health. 2022;8. doi: 10.1177/20552076221116772 PMC9380417 35983102

[23] Yen PY, Wantland D, Bakken S. Development of a customizable health IT usability evaluation scale. AMIA Annu Symp Proc. 2010;2010: 917. https://pmc.ncbi.nlm.nih.gov/articles/PMC3041285/ Accessed 2025 Feb 28. 21347112 PMC3041285

[24] Bonett DG. Sample size requirements for estimating intraclass correlations with desired precision. Stat Med. 2002;21(9):1331-35. doi: 10.1002/sim.1108 12111881

