# Supplementary material for: Feasibility and acceptability of smartphone technology for patients to self-record vital signs in the emergency department (The FacED Study): A study protocol
Source: PLoS One. 2026 Jun 30;21(6):e0326013. doi: 10.1371/journal.pone.0326013 (PMC13318001; doi:10.1371/journal.pone.0326013)
Supplement: Attachment — Submitted filename: PONE-D-25-25645Response to reviewers.docx [file pone.0326013.s002.docx]

**Feasibility and acceptability of smartphone technology for patients to self-record vital signs in the emergency department (The FacED Study): a study protocol**

Response to reviewers.

We are grateful to the reviewers for the time spent with our manuscript. We have revised the content considering reviewer comments where we have retained the content in this shortened version of the results.

*Editorial comments*

| **no** | **Comment** | **Response** |
| --- | --- | --- |
| 1 | Compliance with style and formatting guidance for submission | Apologies. All formatting has been corrected throughout the manuscript to align with journal style guidelines. No tracked changes were retained for formatting amendments, as requested. |
| 2 | Please provide additional details regarding participant consent. In the ethics statement in the Methods and online submission information, please ensure that you have specified (1) whether consent was informed and (2) what type you obtained (for instance, written or verbal, and if verbal, how it was documented and witnessed). | We have revised the Methods and Ethics sections to clarify (1) that informed consent was obtained for all study procedures and (2) the type of consent used. Written informed consent was obtained for WP1, while WP2 and WP3 used self‑completed electronic consent procedures embedded within the survey platforms. |
| 3 | Please state what role the funders took in the study. If the funders had no role, please state: "The funders had no role in study design, data collection and analysis, decision to publish, or preparation of the manuscript." If this statement is not correct you must amend it as needed. Please include this amended Role of Funder statement in your cover letter; we will change the online submission form on your behalf. | We have updated the Funding Statement to specify that the funder had no role in study design, data collection and analysis, decision to publish, or preparation of the manuscript. |
| 4 | Please provide an amended Funding Statement declaring this commercial affiliation, as well as a statement regarding the Role of Funders in your study. If the funding organization did not play a role in the study design, data collection and analysis, decision to publish, or preparation of the manuscript and only provided financial support in the form of authors' salaries and/or research materials, please review your statements relating to the author contributions, and ensure you have specifically and accurately indicated the role(s) that these authors had in your study. You can update author roles in the Author Contributions section of the online submission form. Please also include the following statement within your amended Funding Statement. “The funder provided support in the form of salaries for authors [insert relevant initials], but did not have any additional role in the study design, data collection and analysis, decision to publish, or preparation of the manuscript. The specific roles of these authors are articulated in the ‘author contributions’ section.” If your commercial affiliation did play a role in your study, please state and explain this role within your updated Funding Statement.  b. Please also provide an updated Competing Interests Statement declaring this commercial affiliation along with any other relevant declarations relating to employment, consultancy, patents, products in development, or marketed products, etc. Within your Competing Interests Statement, please confirm that this commercial affiliation does not alter your adherence to all PLOS ONE policies on sharing data and materials by including the following statement: "This does not alter our adherence to PLOS ONE policies on sharing data and materials.” (as detailed online in our guide for authors http://journals.plos.org/plosone/s/competing-interests) . If this adherence statement is not accurate and there are restrictions on sharing of data and/or materials, please state these. Please note that we cannot proceed with consideration of your article until this information has been declared. Please include both an updated Funding Statement and Competing Interests Statement in your cover letter. We will change the online submission form on your behalf. | - Commercial Affiliation had no role in study design, data collection and analysis, decision to publish, or preparation of the manuscript - We have updated the Funding Statement to specify that the funder had no role in study design, data collection and analysis, decision to publish, or preparation of the manuscript. |
| 5 | Your ethics statement should only appear in the Methods section of your manuscript. | The ethics statement has been removed from the Abstract and retained only within the dedicated Ethics section, in line with journal guidelines. |

*Reviewer 1*

| **no** | **Comment** | **Response** |
| --- | --- | --- |
| 1 | - l. 81: I did not understand how a self-measurement is supposed to adequately monitor clinical deterioration. | Self‑measurement is not intended to replace clinical assessment or structured monitoring pathways. Instead, early patient‑led data capture may reduce delays to obtaining baseline vital signs during periods of crowding and will provide clinicians with earlier visibility of physiological status while patients await formal triage. |
| 2 | 126: absence of serious medical condition and l 138 Clinical condition warranting immediate medical attention need to be opjective. How do you assess these criteria (i.e. triage categories...)? Please elaborate to make sure that it is clear which population is to be recruited. | Exclusion criteria has been updated to clarify use of Manchester Triage Categories. |
| 3 | 140 f.: Why do you use the ICC for sample size calculation and how does this reflect your research objective? It seems that your research question might be more related to: 10.1515/ijb-2015-0039. It might also be interesting to look at it from a clinical perspective and see how sensitivity and specificity compare for the different measurements for all vital signs under consideration. Please also add a clear hypothesis you are testing. | The study size section for WP1 has been expanded to provide more detail and justification for the sample size. The ICC is selected as the appropriate measure of agreement because it quantifies concordance between two continuous measurement methods taken under near‑identical conditions. This study will treat performance assessment as a secondary exploratory outcome. |
| 4 | - l. 163: How do you make sure that measurements are comparable between different health care professionals and which measurements will you take from the standard monitor (when, how many)? Maybe you can elaborate that more in the bias chapter (l. 217) or in the data collection section. | The timings and recording for the contemporaneous recording have been added in the text |
| 5 | l. 167: Does that include reduction of crowding, better utilization of resources, better recording of vital signs (i.e. less missing data)? | We agree that reductions in crowding, improved resource utilisation, and improved completeness of vital‑sign documentation are potential downstream benefits of patient‑recorded vital signs. However, this feasibility study is not designed or powered to measure these outcomes. We have included clarification of this in the A&O |
| 6 | l. 175: How can the different measurements be attributed to the same person via this data collection mechanism? | The timings and recording for the contemporaneous recording have been added in the text |
| 7 | - l. 175: Does this reflect routine care for later implementation of the tool? What biases might this entail? | Vital sign measures are being recorded by research nurses alongside the patient recording their own vital signs. |
| 8 | l. 222f: How do you assess the influence of the center in the analysis? | We are not formally modelling the effects of different centers. |
| 9 | l. 235: What are these subgroups and how is that accounted for in the sample size calculation/analysis (multiple testing?)? | Detail on a priori sub analysis has been included in the text |
| 10 | Maybe you can add a short discussion regarding the potential implications for your research project. In this regard, I am wondering if such a system can be easily implemented into the electronic medical records. You could also mention the strengths and limitations from the abstract. - | We added a short paragraph to the Discussion summarising potential implications and feasibility of EMR integration. |
| 11 | Can you further elaborate if the datasets are made available? At least it is stated on page 7. This should also be in the manuscript itself. | Study datasets will be made available upon reasonable request to the corresponding author, in accordance with institutional data‑sharing policies. |

*Reviewer 2*

| **no** | **Comment** | **Response** |
| --- | --- | --- |
| 1 | While feasibility and acceptability are clearly stated as the main aims, the performance/accuracy assessment using ICC appears methodologically substantial and may be interpreted by readers as a co-primary outcome. | We added wording clarifying that performance (ICC) is a secondary exploratory outcome and not co‑primary. |
| 2 | Feasibility is defined narrowly as the proportion of patients able to complete the recording | We have expanded the definition to include completeness of recordings, ability to engage with instructions, and whether assistance was required. |
| 3 | The protocol appropriately acknowledges exclusion of non-English-speaking participants, but the impact on equity is underdeveloped | We added a paragraph acknowledging implications for digital exclusion and equity. |
| 4 | The protocol states that vital signs will be measured “near-simultaneously” using standard methods | The timings for the contemporaneous recording have been added in the text |
| 5 | Inclusion of the Fitzpatrick skin tone scale is a major strength, but its analytical role is not clearly specified. | We have added the explanation that Fitzpatrick data will be used for subgroup ICC analyses only. |
| 6 | A few grammatical issues (e.g., “patients’ presenting with illness conditions”). Occasional spacing and punctuation inconsistencies in bullet lists | Thank you, grammatical issues have been resolved and formatted as per paper guidelines. |
