# Supplementary material for: Feasibility and acceptability of smartphone technology for patients to self-record vital signs in the emergency department (The FacED Study): A study protocol
Source: PLoS One. 2026 Jun 30;21(6):e0326013. doi: 10.1371/journal.pone.0326013 (PMC13318001; doi:10.1371/journal.pone.0326013)
Supplement: Peer review history

# Peer review: Decision Letter 0

**DOI:** 10.1371/journal.pone.0326013.r001

**Authors:** Haipeng Liu

16 Feb 2026

PONE-D-25-25645

Feasibility and acceptability of smartphone technology for patients to self-record vital signs in the emergency department (The FacED Study): a study protocol

PLOS One

Dear Dr. Jarman,

Thank you for submitting your manuscript to PLOS ONE. After careful consideration, we feel that it has merit but does not fully meet PLOS ONE’s publication criteria as it currently stands. Therefore, we invite you to submit a revised version of the manuscript that addresses the points raised during the review process.

Please submit your revised manuscript by Apr 02 2026 11:59PM. If you will need more time than this to complete your revisions, please reply to this message or contact the journal office at plosone@plos.org. When you're ready to submit your revision, log on to https://www.editorialmanager.com/pone/ and select the 'Submissions Needing Revision' folder to locate your manuscript file.

Please include the following items when submitting your revised manuscript:

- A letter that responds to each point raised by the academic editor and reviewer(s). You should upload this letter as a separate file labeled 'Response to Reviewers'.
- A marked-up copy of your manuscript that highlights changes made to the original version. You should upload this as a separate file labeled 'Revised Manuscript with Track Changes'.
- An unmarked version of your revised paper without tracked changes. You should upload this as a separate file labeled 'Manuscript'.

If you would like to make changes to your financial disclosure, please include your updated statement in your cover letter. Guidelines for resubmitting your figure files are available below the reviewer comments at the end of this letter.

If applicable, we recommend that you deposit your laboratory protocols in protocols.io to enhance the reproducibility of your results. Protocols.io assigns your protocol its own identifier (DOI) so that it can be cited independently in the future. For instructions see: https://journals.plos.org/plosone/s/submission-guidelines#loc-laboratory-protocols. Additionally, PLOS ONE offers an option for publishing peer-reviewed Lab Protocol articles, which describe protocols hosted on protocols.io. Read more information on sharing protocols at https://plos.org/protocols?utm_medium=editorial-email&utm_source=authorletters&utm_campaign=protocols.

We look forward to receiving your revised manuscript.

Kind regards,

Haipeng Liu

Academic Editor

PLOS One

Journal Requirements:

When submitting your revision, we need you to address these additional requirements.

1. Please ensure that your manuscript meets PLOS ONE's style requirements, including those for file naming. The PLOS ONE style templates can be found at

https://journals.plos.org/plosone/s/file?id=wjVg/PLOSOne_formatting_sample_main_body.pdf and

https://journals.plos.org/plosone/s/file?id=ba62/PLOSOne_formatting_sample_title_authors_affiliations.pdf

2. Please provide additional details regarding participant consent. In the ethics statement in the Methods and online submission information, please ensure that you have specified (1) whether consent was informed and (2) what type you obtained (for instance, written or verbal, and if verbal, how it was documented and witnessed). If your study included minors, state whether you obtained consent from parents or guardians. If the need for consent was waived by the ethics committee, please include this information.

If you are reporting a retrospective study of medical records or archived samples, please ensure that you have discussed whether all data were fully anonymized before you accessed them and/or whether the IRB or ethics committee waived the requirement for informed consent. If patients provided informed written consent to have data from their medical records used in research, please include this information.

3. Thank you for stating the following financial disclosure:

This work was awarded by the Royal College of Emergency Medicine (RCEM) Autumn 2024 grant to JCW. https://rcem.ac.uk/ The funder played no role in the study design or publication decision.

Please state what role the funders took in the study. If the funders had no role, please state: "The funders had no role in study design, data collection and analysis, decision to publish, or preparation of the manuscript."

If this statement is not correct you must amend it as needed.

Please include this amended Role of Funder statement in your cover letter; we will change the online submission form on your behalf.

4. Thank you for stating the following in the Competing Interests/Financial Disclosure section:

I have read the journal's policy and the authors of this manuscript have the following competing interests:

Dr Gabriel Jones: Unpaid role as medical officer at streamwave.ai. This may translate into shares or a paid position in the future.

We note that one or more of the authors are employed by a commercial company: streamwave.ai

a. Please provide an amended Funding Statement declaring this commercial affiliation, as well as a statement regarding the Role of Funders in your study. If the funding organization did not play a role in the study design, data collection and analysis, decision to publish, or preparation of the manuscript and only provided financial support in the form of authors' salaries and/or research materials, please review your statements relating to the author contributions, and ensure you have specifically and accurately indicated the role(s) that these authors had in your study. You can update author roles in the Author Contributions section of the online submission form.

Please also include the following statement within your amended Funding Statement.

“The funder provided support in the form of salaries for authors [insert relevant initials], but did not have any additional role in the study design, data collection and analysis, decision to publish, or preparation of the manuscript. The specific roles of these authors are articulated in the ‘author contributions’ section.”

If your commercial affiliation did play a role in your study, please state and explain this role within your updated Funding Statement.

b. Please also provide an updated Competing Interests Statement declaring this commercial affiliation along with any other relevant declarations relating to employment, consultancy, patents, products in development, or marketed products, etc.

Within your Competing Interests Statement, please confirm that this commercial affiliation does not alter your adherence to all PLOS ONE policies on sharing data and materials by including the following statement: "This does not alter our adherence to PLOS ONE policies on sharing data and materials.” (as detailed online in our guide for authors http://journals.plos.org/plosone/s/competing-interests) . If this adherence statement is not accurate and there are restrictions on sharing of data and/or materials, please state these. Please note that we cannot proceed with consideration of your article until this information has been declared.

Please include both an updated Funding Statement and Competing Interests Statement in your cover letter. We will change the online submission form on your behalf.

5. Your ethics statement should only appear in the Methods section of your manuscript. If your ethics statement is written in any section besides the Methods, please move it to the Methods section and delete it from any other section. Please ensure that your ethics statement is included in your manuscript, as the ethics statement entered into the online submission form will not be published alongside your manuscript.

6. If the reviewer comments include a recommendation to cite specific previously published works, please review and evaluate these publications to determine whether they are relevant and should be cited. There is no requirement to cite these works unless the editor has indicated otherwise.

Additional Editor Comments:

The reviewers provides some suggestions on the methodological details. Please revise.

[Note: HTML markup is below. Please do not edit.]

Reviewers' comments:

Reviewer's Responses to Questions

**Comments to the Author**

1. Does the manuscript provide a valid rationale for the proposed study, with clearly identified and justified research questions?

The research question outlined is expected to address a valid academic problem or topic and contribute to the base of knowledge in the field.

Reviewer #1: Yes

Reviewer #2: Yes

**********

2. Is the protocol technically sound and planned in a manner that will lead to a meaningful outcome and allow testing the stated hypotheses?

The manuscript should describe the methods in sufficient detail to prevent undisclosed flexibility in the experimental procedure or analysis pipeline, including sufficient outcome-neutral conditions (e.g. necessary controls, absence of floor or ceiling effects) to test the proposed hypotheses and a statistical power analysis where applicable. As there may be aspects of the methodology and analysis which can only be refined once the work is undertaken, authors should outline potential assumptions and explicitly describe what aspects of the proposed analyses, if any, are exploratory.

Reviewer #1: Partly

Reviewer #2: Yes

**********

3. Is the methodology feasible and described in sufficient detail to allow the work to be replicable?

Descriptions of methods and materials in the protocol should be reported in sufficient detail for another researcher to reproduce all experiments and analyses. The protocol should describe the appropriate controls, sample size calculations, and replication needed to ensure that the data are robust and reproducible.

Reviewer #1: Yes

Reviewer #2: Yes

**********

4. Have the authors described where all data underlying the findings will be made available when the study is complete?

The PLOS Data policy requires authors to make all data underlying the findings described in their manuscript fully available without restriction, with rare exception, at the time of publication. The data should be provided as part of the manuscript or its supporting information, or deposited to a public repository. For example, in addition to summary statistics, the data points behind means, medians and variance measures should be available. If there are restrictions on publicly sharing data—e.g. participant privacy or use of data from a third party—those must be specified.

Reviewer #1: No

Reviewer #2: Yes

**********

5. Is the manuscript presented in an intelligible fashion and written in standard English?

PLOS ONE does not copyedit accepted manuscripts, so the language in submitted articles must be clear, correct, and unambiguous. Any typographical or grammatical errors should be corrected at revision, so please note any specific errors here.

Reviewer #1: Yes

Reviewer #2: Yes

**********

6. Review Comments to the Author

Please use the space provided to explain your answers to the questions above and, if applicable, provide comments about issues authors must address before this protocol can be accepted for publication. You may also include additional comments for the author, including concerns about research or publication ethics.

You may also provide optional suggestions and comments to authors that they might find helpful in planning their study.

(Please upload your review as an attachment if it exceeds 20,000 characters)

Reviewer #1: Thank you very much for the opportunity to read this interesting protocol of an observational study on self-recorded vital signs via smartphone. The study comprises several work packages and seeks to analyse the performance of the method compared to standard measurements via monitor, clinical acceptability, as well as a cross-sectional survey on interest in mhealth technologies.

The overall protocol is concise and thorough using the STROBE statement. I have several comments that would make it easier for me to fully understand all relevant aspects of the project.

The following comments should be adressed:

- l. 81: I did not understand how a self-measurement is supposed to adequately monitor clinical deterioration.

- l. 126: absence of serious medical condition and l 138 Clinical condition warranting immediate medical attention need to be opjective. How do you assess these criteria (i.e. triage categories...)? Please elaborate to make sure that it is clear which population is to be recruited.

- l. 140 f.: Why do you use the ICC for sample size calculation and how does this reflect your research objective? It seems that your research question might be more related to: 10.1515/ijb-2015-0039. It might also be interesting to look at it from a clinical perspective and see how sensitivity and specificity compare for the different measurements for all vital signs under consideration. Please also add a clear hypothesis you are testing.

- l. 163: How do you make sure that measurements are comparable between different health care professionals and which measurements will you take from the standard monitor (when, how many)? Maybe you can elaborate that more in the bias chapter (l. 217) or in the data collection section.

- l. 167: Does that include reduction of crowding, better utilization of resources, better recording of vital signs (i.e. less missing data)?

- l. 175: How can the different measurements be attributed to the same person via this data collection mechanism?

- l. 175: Does this reflect routine care for later implementation of the tool? What biases might this entail?

- l. 222f: How do you assess the influcence of the center in the analysis?

- l. 235: What are these subgroups and how is that accounted for in the sample size calculation/analysis (multiple testing?)?

- Maybe you can add a short discussion regarding the potential implications for your research project. In this regard, I am wondering if such a system can be easily implemented into the electronic medical records. You could also mention the strengths and limitations from the abstract.

- Can you further elaborate if the datasets are made available? At least it is stated on page 7. This should also be in the manuscript itself.

Reviewer #2: This is a well-written, timely, and methodologically sound study protocol addressing an important operational challenge in urgent and emergency care: incomplete or delayed vital sign measurement due to overcrowding and nursing workload. The protocol is clearly structured, ethically approved, and appropriately powered for its primary feasibility and performance objectives. The multicenter design within a single NHS Trust enhances internal consistency while still allowing diversity in care settings. The study is suitable for publication as a protocol, with minor revisions recommended to improve clarity, transparency, and reproducibility.

1. While feasibility and acceptability are clearly stated as the main aims, the performance/accuracy assessment using ICC appears methodologically substantial and may be interpreted by readers as a co-primary outcome.

2. Feasibility is defined narrowly as the proportion of patients able to complete the recording.

3. The protocol appropriately acknowledges exclusion of non-English-speaking participants, but the impact on equity is underdeveloped.

4. The protocol states that vital signs will be measured “near-simultaneously” using standard methods.

5. Inclusion of the Fitzpatrick skin tone scale is a major strength, but its analytical role is not clearly specified.

6. A few grammatical issues (e.g., “patients’ presenting with illness conditions”). Occasional spacing and punctuation inconsistencies in bullet lists.

This protocol describes a robust and highly relevant feasibility study with clear clinical and operational implications for emergency care. With minor revisions, particularly around outcome hierarchy, feasibility definition, and subgroup analyses, the manuscript is well suited for publication and will provide a strong foundation for subsequent effectiveness studies

**********

7. PLOS authors have the option to publish the peer review history of their article (what does this mean?). If published, this will include your full peer review and any attached files.

If you choose “no”, your identity will remain anonymous but your review may still be made public.

**Do you want your identity to be public for this peer review?**  For information about this choice, including consent withdrawal, please see our Privacy Policy.

Reviewer #1: **Yes:**Hendrik Napierala

Reviewer #2: **Yes:**Faizan Akram

**********

[NOTE: If reviewer comments were submitted as an attachment file, they will be attached to this email and accessible via the submission site. Please log into your account, locate the manuscript record, and check for the action link "View Attachments". If this link does not appear, there are no attachment files.]

To ensure your figures meet our technical requirements, please review our figure guidelines: https://journals.plos.org/plosone/s/figures

You may also use PLOS’s free figure tool, NAAS, to help you prepare publication quality figures: https://journals.plos.org/plosone/s/figures#loc-tools-for-figure-preparation.

NAAS will assess whether your figures meet our technical requirements by comparing each figure against our figure specifications.

# Author comment: Author response to Decision Letter 1

**DOI:** 10.1371/journal.pone.0326013.r002

14 Apr 2026

We are grateful to the reviewers for the time spent with our manuscript. We have revised the content considering reviewer comments where we have retained the content in this shortened version of the results.

Editorial comments

Compliance with style and formatting guidance for submission

Apologies. All formatting has been corrected throughout the manuscript to align with journal style guidelines. No tracked changes were retained for formatting amendments, as requested.

Please provide additional details regarding participant consent. In the ethics statement in the Methods and online submission information, please ensure that you have specified (1) whether consent was informed and (2) what type you obtained (for instance, written or verbal, and if verbal, how it was documented and witnessed).

We have revised the Methods and Ethics sections to clarify (1) that informed consent was obtained for all study procedures and (2) the type of consent used. Written informed consent was obtained for WP1, while WP2 and WP3 used self‑completed electronic consent procedures embedded within the survey platforms.

Please state what role the funders took in the study. If the funders had no role, please state: "The funders had no role in study design, data collection and analysis, decision to publish, or preparation of the manuscript." If this statement is not correct you must amend it as needed. Please include this amended Role of Funder statement in your cover letter; we will change the online submission form on your behalf.

We have updated the Funding Statement to specify that the funder had no role in study design, data collection and analysis, decision to publish, or preparation of the manuscript.

Please provide an amended Funding Statement declaring this commercial affiliation, as well as a statement regarding the Role of Funders in your study. If the funding organization did not play a role in the study design, data collection and analysis, decision to publish, or preparation of the manuscript and only provided financial support in the form of authors' salaries and/or research materials, please review your statements relating to the author contributions, and ensure you have specifically and accurately indicated the role(s) that these authors had in your study. You can update author roles in the Author Contributions section of the online submission form. Please also include the following statement within your amended Funding Statement. “The funder provided support in the form of salaries for authors [insert relevant initials], but did not have any additional role in the study design, data collection and analysis, decision to publish, or preparation of the manuscript. The specific roles of these authors are articulated in the ‘author contributions’ section.” If your commercial affiliation did play a role in your study, please state and explain this role within your updated Funding Statement.

b. Please also provide an updated Competing Interests Statement declaring this commercial affiliation along with any other relevant declarations relating to employment, consultancy, patents, products in development, or marketed products, etc. Within your Competing Interests Statement, please confirm that this commercial affiliation does not alter your adherence to all PLOS ONE policies on sharing data and materials by including the following statement: "This does not alter our adherence to PLOS ONE policies on sharing data and materials.” (as detailed online in our guide for authors http://journals.plos.org/plosone/s/competing-interests) . If this adherence statement is not accurate and there are restrictions on sharing of data and/or materials, please state these. Please note that we cannot proceed with consideration of your article until this information has been declared. Please include both an updated Funding Statement and Competing Interests Statement in your cover letter. We will change the online submission form on your behalf.

Commercial Affiliation had no role in study design, data collection and analysis, decision to publish, or preparation of the manuscript

We have updated the Funding Statement to specify that the funder had no role in study design, data collection and analysis, decision to publish, or preparation of the manuscript.

Your ethics statement should only appear in the Methods section of your manuscript.

The ethics statement has been removed from the Abstract and retained only within the dedicated Ethics section, in line with journal guidelines.

Reviewer 1

- l. 81: I did not understand how a self-measurement is supposed to adequately monitor clinical deterioration.

Self‑measurement is not intended to replace clinical assessment or structured monitoring pathways. Instead, early patient‑led data capture may reduce delays to obtaining baseline vital signs during periods of crowding and will provide clinicians with earlier visibility of physiological status while patients await formal triage.

126: absence of serious medical condition and l 138 Clinical condition warranting immediate medical attention need to be opjective. How do you assess these criteria (i.e. triage categories...)? Please elaborate to make sure that it is clear which population is to be recruited.

Exclusion criteria has been updated to clarify use of Manchester Triage Categories.

140 f.: Why do you use the ICC for sample size calculation and how does this reflect your research objective? It seems that your research question might be more related to: 10.1515/ijb-2015-0039. It might also be interesting to look at it from a clinical perspective and see how sensitivity and specificity compare for the different measurements for all vital signs under consideration. Please also add a clear hypothesis you are testing.

The study size section for WP1 has been expanded to provide more detail and justification for the sample size. The ICC is selected as the appropriate measure of agreement because it quantifies concordance between two continuous measurement methods taken under near‑identical conditions. This study will treat performance assessment as a secondary exploratory outcome.

- l. 163: How do you make sure that measurements are comparable between different health care professionals and which measurements will you take from the standard monitor (when, how many)? Maybe you can elaborate that more in the bias chapter (l. 217) or in the data collection section.

The timings and recording for the contemporaneous recording have been added in the text

l. 167: Does that include reduction of crowding, better utilization of resources, better recording of vital signs (i.e. less missing data)?

We agree that reductions in crowding, improved resource utilisation, and improved completeness of vital‑sign documentation are potential downstream benefits of patient‑recorded vital signs. However, this feasibility study is not designed or powered to measure these outcomes. We have included clarification of this in the A&O

l. 175: How can the different measurements be attributed to the same person via this data collection mechanism?

The timings and recording for the contemporaneous recording have been added in the text

l. 175: Does this reflect routine care for later implementation of the tool? What biases might this entail?

Vital sign measures are being recorded by research nurses alongside the patient recording their own vital signs.

l. 222f: How do you assess the influence of the center in the analysis?

We are not formally modelling the effects of different centers.

l. 235: What are these subgroups and how is that accounted for in the sample size calculation/analysis (multiple testing?)?

Detail on a priori sub analysis has been included in the text

Maybe you can add a short discussion regarding the potential implications for your research project. In this regard, I am wondering if such a system can be easily implemented into the electronic medical records. You could also mention the strengths and limitations from the abstract. -

We added a short paragraph to the Discussion summarising potential implications and feasibility of EMR integration.

Can you further elaborate if the datasets are made available? At least it is stated on page 7. This should also be in the manuscript itself.

Study datasets will be made available upon reasonable request to the corresponding author, in accordance with institutional data‑sharing policies.

Reviewer 2

While feasibility and acceptability are clearly stated as the main aims, the performance/accuracy assessment using ICC appears methodologically substantial and may be interpreted by readers as a co-primary outcome.

We added wording clarifying that performance (ICC) is a secondary exploratory outcome and not co‑primary.

Feasibility is defined narrowly as the proportion of patients able to complete the recording

We have expanded the definition to include completeness of recordings, ability to engage with instructions, and whether assistance was required.

The protocol appropriately acknowledges exclusion of non-English-speaking participants, but the impact on equity is underdeveloped

We added a paragraph acknowledging implications for digital exclusion and equity.

The protocol states that vital signs will be measured “near-simultaneously” using standard methods

The timings for the contemporaneous recording have been added in the text

Inclusion of the Fitzpatrick skin tone scale is a major strength, but its analytical role is not clearly specified.

We have added the explanation that Fitzpatrick data will be used for subgroup ICC analyses only.

A few grammatical issues (e.g., “patients’ presenting with illness conditions”). Occasional spacing and punctuation inconsistencies in bullet lists

Thank you, grammatical issues have been resolved and formatted as per paper guidelines.

Attachment Submitted filename: PONE-D-25-25645Response to reviewers.docx

# Peer review: Decision Letter 1

**DOI:** 10.1371/journal.pone.0326013.r003

**Authors:** Haipeng Liu

1 May 2026

Feasibility and acceptability of smartphone technology for patients to self-record vital signs in the emergency department (The FacED Study): a study protocol

PONE-D-25-25645R1

Dear Dr. Jarman,

We’re pleased to inform you that your manuscript has been judged scientifically suitable for publication and will be formally accepted for publication once it meets all outstanding technical requirements.

Within one week, you’ll receive an e-mail detailing the required amendments. When these have been addressed, you’ll receive a formal acceptance letter and your manuscript will be scheduled for publication.

An invoice will be generated when your article is formally accepted. Please note, if your institution has a publishing partnership with PLOS and your article meets the relevant criteria, all or part of your publication costs will be covered. Please make sure your user information is up-to-date by logging into Editorial Manager at Editorial Manager® and clicking the ‘Update My Information' link at the top of the page. For questions related to billing, please contact billing support.

If your institution or institutions have a press office, please notify them about your upcoming paper to help maximize its impact. If they’ll be preparing press materials, please inform our press team as soon as possible -- no later than 48 hours after receiving the formal acceptance. Your manuscript will remain under strict press embargo until 2 pm Eastern Time on the date of publication. For more information, please contact onepress@plos.org.

Kind regards,

Haipeng Liu

Academic Editor

PLOS One

Additional Editor Comments (optional):

Reviewers' comments:

Reviewer's Responses to Questions

**Comments to the Author**

1. Does the manuscript provide a valid rationale for the proposed study, with clearly identified and justified research questions?

The research question outlined is expected to address a valid academic problem or topic and contribute to the base of knowledge in the field.

Reviewer #1: Yes

Reviewer #2: Yes

**********

2. Is the protocol technically sound and planned in a manner that will lead to a meaningful outcome and allow testing the stated hypotheses?

The manuscript should describe the methods in sufficient detail to prevent undisclosed flexibility in the experimental procedure or analysis pipeline, including sufficient outcome-neutral conditions (e.g. necessary controls, absence of floor or ceiling effects) to test the proposed hypotheses and a statistical power analysis where applicable. As there may be aspects of the methodology and analysis which can only be refined once the work is undertaken, authors should outline potential assumptions and explicitly describe what aspects of the proposed analyses, if any, are exploratory.

Reviewer #1: Yes

Reviewer #2: Yes

**********

3. Is the methodology feasible and described in sufficient detail to allow the work to be replicable?

Descriptions of methods and materials in the protocol should be reported in sufficient detail for another researcher to reproduce all experiments and analyses. The protocol should describe the appropriate controls, sample size calculations, and replication needed to ensure that the data are robust and reproducible.

Reviewer #1: Yes

Reviewer #2: Yes

**********

4. Have the authors described where all data underlying the findings will be made available when the study is complete?

The PLOS Data policy requires authors to make all data underlying the findings described in their manuscript fully available without restriction, with rare exception, at the time of publication. The data should be provided as part of the manuscript or its supporting information, or deposited to a public repository. For example, in addition to summary statistics, the data points behind means, medians and variance measures should be available. If there are restrictions on publicly sharing data—e.g. participant privacy or use of data from a third party—those must be specified.

Reviewer #1: Yes

Reviewer #2: Yes

**********

5. Is the manuscript presented in an intelligible fashion and written in standard English?

PLOS ONE does not copyedit accepted manuscripts, so the language in submitted articles must be clear, correct, and unambiguous. Any typographical or grammatical errors should be corrected at revision, so please note any specific errors here.

Reviewer #1: Yes

Reviewer #2: Yes

**********

6. Review Comments to the Author

Please use the space provided to explain your answers to the questions above and, if applicable, provide comments about issues authors must address before this protocol can be accepted for publication. You may also include additional comments for the author, including concerns about research or publication ethics.

You may also provide optional suggestions and comments to authors that they might find helpful in planning their study.

(Please upload your review as an attachment if it exceeds 20,000 characters)

Reviewer #1: Thank you very much for the careful revision of your manuscript. All my comments were adressed. Therefore, I can recommend publication of the manuscript.

Reviewer #2: The study has the potential to contribute significantly and the article is suitable for publication.

**********

7. PLOS authors have the option to publish the peer review history of their article (what does this mean?). If published, this will include your full peer review and any attached files.

If you choose “no”, your identity will remain anonymous but your review may still be made public.

**Do you want your identity to be public for this peer review?**  For information about this choice, including consent withdrawal, please see our Privacy Policy.

Reviewer #1: **Yes:**Hendrik Napierala

Reviewer #2: **Yes:**Faizan Akram

**********

# Editor report: Acceptance letter

**DOI:** 10.1371/journal.pone.0326013.r004

**Authors:** Haipeng Liu

PONE-D-25-25645R1

PLOS One

Dear Dr. Jarman,

I'm pleased to inform you that your manuscript has been deemed suitable for publication in PLOS One. Congratulations! Your manuscript is now being handed over to our production team.

At this stage, our production department will prepare your paper for publication. This includes ensuring the following:

* All references, tables, and figures are properly cited

* All relevant supporting information is included in the manuscript submission,

* There are no issues that prevent the paper from being properly typeset

You will receive further instructions from the production team, including instructions on how to review your proof when it is ready. Please keep in mind that we are working through a large volume of accepted articles, so please give us a few days to review your paper and let you know the next and final steps.

Lastly, if your institution or institutions have a press office, please let them know about your upcoming paper now to help maximize its impact. If they'll be preparing press materials, please inform our press team within the next 48 hours. Your manuscript will remain under strict press embargo until 2 pm Eastern Time on the date of publication. For more information, please contact onepress@plos.org.

You will receive an invoice from PLOS for your publication fee after your manuscript has reached the completed accept phase. If you receive an email requesting payment before acceptance or for any other service, this may be a phishing scheme. Learn how to identify phishing emails and protect your accounts at https://explore.plos.org/phishing.

If we can help with anything else, please email us at customercare@plos.org.

Thank you for submitting your work to PLOS ONE and supporting open access.

Kind regards,

PLOS ONE Editorial Office Staff

on behalf of

Dr. Haipeng Liu

Academic Editor

PLOS One
